# Recent Work on Artificial Pneumothorax

**Published:** 1917-04-14

**Authors:** 


### RECENT WORK ON ARTIFICIAL PNEUMOTHORAX.

### Benefits and their Limitations.

The latest number of the *Internationales Centralblatt für die gesamte Tuberkulose-Forschung* to reach this country is a special number (the tenth) devoted to literature on lung collapse therapeutics. Schröder has a paper on artificial pneumothorax induction as an aid to diagnosis of pulmonary tumours. Following, as others have done, a suggestion of Brauer's, he gives two cases of chronic lung disease diagnosed as tuberculous where the presence of a pleural effusion made the *x*-ray picture of no avail. When, however, the fluid was evacuated by thoracocentesis and replaced with nitrogen gas, the skiagraph showed a lung tumour plainly standing out against the clear pneumothorax and the collapsed normal pulmonary tissue. So far from this diagnostic procedure being injurious to the patient, it was found to be actually palliative, the compression of the cancerous lung leading to an abatement of symptoms a little as if it had been phthisical. This therapeutic effect was, of course, only transitory.

### Air *versus* Nitrogen.

From Davos come some investigations as to the fate of the gas introduced into the pleural space. Other inquiries have resulted in the conclusion that no matter whether nitrogen alone or ordinary air is used, the "residual" gas, *i.e.* that present in the chest a week or so after the filling, is a mixture containing both oxygen and carbon dioxide. These findings have helped to discredit the use of pure nitrogen instead of air; the latter is also preferable for convenience' sake and economy. The authors describe the residual gas mixture as being of oxygen, nitrogen dioxide, and carbon dioxide, in proportions pretty constant in different patients. The presence of an effusion lowers, however, the amount of oxygen present. Most of the above gaseous change must be from the interaction of the tissues and the pneumothorax. They make the suggestion that the permanence of an artificial pneumothorax (and therefore the intervals of time between "refills") might possibly be increased by using this final mixture of gases to induce the pneumothorax with, and to keep it up.

### Some Inconclusive Experiments.

In a Viennese medical paper are reported the results, in dogs, of tying the pulmonary artery with the object of bringing on fibrosis of the lung, the aim, or partial aim, of all phthisio-therapeutics. In this animal regressive changes were at first caused, even amounting to necrosis, especially in the deeper parts of the lungs; in the more superficial, sub-pleural portions they were less noticeable. Later, fibrous tissue was developed. The writers consider that their results argue a possible clinical significance for human patients. One would think, however, that any therapeutic effect should first be tested on animals actually phthisical.

Gradí (*Le pneumothorax Thérapeutique*, Bd. II., No. 1) recommends artificial pneumothorax as a help in the radiological investigation of chest wounds, just as Schröder did in lung cancer. The relation of a bullet to surrounding structures can thus be well ascertained, whilst there is likewise a therapeutic effect in quicker healing of the lung when compressed and relatively at rest.

### A Discussion in Norway.

The discussion on artificial pneumothorax treatment at a meeting of the Christiania Medical Society revealed some of the limitations of this method. It should be borne in mind, however, that lung compression is mostly applicable in severe lesions that have resisted other treatment, and is thus in quite another category of usefulness from some medicament or other that is given to early cases simultaneously with a hygienic-dietetic routine, the good effects of which last are largely claimed for the specific remedy employed. Holmboe (Mesnalien) reported that since 1907—a date years subsequent to which no case had been treated thus in Great Britain—artificial pneumothorax had been tried in sixty-three patients, or 12 per cent. of the total number admitted. Of the sixty-three only two were in the first, and no less than forty-three in the third, stage of phthisis. Forty-three, too, was the number in which gas was got into the pleura; and of these, nine were free from symptoms seven years after the close of treatment, nine were still under treatment, and twenty-five were dead. A pulmonary exudation occurred in twenty-two, or 50 per cent.

### A Kill-or-Cure Method.

Tillisch (Grefsen public sanatorium) in six years had treated seventy-six patients, succeeding in establishing an air space in fifty-three, of which last six were cured, eight improved, twenty-nine dead, and ten still under treatment. Nicolaysen has discontinued pneumothorax work, finding, indeed, like all others, no dangerous effects, but being sceptical of its good results, and thinking that some cases were made worse. Bull has used extra-pleural thoracoplasty on eight very advanced cases; two were certainly, and one apparently cured. He is pleased with this form of treatment. Holst agreed with Nicolaysen , but gave no figures. The impression one gets from the discussion goes to confirm an opinion of lung collapse therapy—namely, that it is an affair of (to quote a motto of a famous compatriot of the above gentlemen) "all or none." Its sphere of application is in very severe disease. If it succeeds, the success is signal, rapid, unmistakable. On the other hand, it much oftener fails altogether. But with proper technique the risk to life is almost nil—the immediate risk, that is—while the slight aggravation of the tuberculous process that occasionally occurs is but as dust in the balance of the fate of one ill with progressive phthisis.

